# The Dual Role of TAM Receptors in Autoimmune Diseases and Cancer: An Overview

**DOI:** 10.3390/cells7100166

**Published:** 2018-10-12

**Authors:** Martha Wium, Juliano D. Paccez, Luiz F. Zerbini

**Affiliations:** 1 Cancer Genomics Group, International Centre for Genetic Engineering and Biotechnology, Cape Town 7925, South Africa; mariet.wium@icgeb.org; 2 Instituto de Ciências Biológicas, Universidade Federal de Goiás, Goiânia 74690-900, GO, Brazil; julianopaccez@gmail.com

**Keywords:** autoimmune disease, Axl, cancer, Mer, TAM receptors, Tyro3

## Abstract

Receptor tyrosine kinases (RTKs) regulate cellular processes by converting signals from the extracellular environment to the cytoplasm and nucleus. Tyro3, Axl, and Mer (TAM) receptors form an RTK family that plays an intricate role in tissue maintenance, phagocytosis, and inflammation as well as cell proliferation, survival, migration, and development. Defects in TAM signaling are associated with numerous autoimmune diseases and different types of cancers. Here, we review the structure of TAM receptors, their ligands, and their biological functions. We discuss the role of TAM receptors and soluble circulating TAM receptors in the autoimmune diseases systemic lupus erythematosus (SLE) and multiple sclerosis (MS). Lastly, we discuss the effect of TAM receptor deregulation in cancer and explore the therapeutic potential of TAM receptors in the treatment of diseases.

## 1. Introduction

Receptor tyrosine kinases (RTKs) regulate normal cellular processes, including growth, survival, differentiation, motility, and adhesion by converting signals from the extracellular environment to the cytoplasm and nucleus [1]. The TAM family of RTKs has three members: Tyro3, Axl, and Mer. In mice, TAM receptors have no essential embryonic function, since single-, double-, and even triple-knockout mice are viable, showing no obvious development defects at birth [2]. Conversely, in all adult knockouts (even single knockouts), mice show a multitude of irregularities, indicating an essential role in maintenance and homeostatic balance in a wide variety of mature organ systems that are subjected to renewal throughout adult life. These include the mature immune, hematopoietic, nervous, vascular, and reproductive systems [2].

TAM receptors have been reported to play a role in a broad spectrum of human autoimmune disorders such as rheumatoid arthritis [3], multiple sclerosis (MS) [4], and systemic lupus erythematosus (SLE) [5], as well in cancer (reviewed in [6]). In cancer, overexpression of TAM receptors has been associated with chemo-resistance [7], metastasis [8], and poor survival outcomes [9].

In this review we will discuss the biological function of TAM receptors and their ligands. We explore the deregulation of TAM receptors in the autoimmune diseases and in cancer. We provide a description of the pathways involved in the different diseases, the role of TAM receptor deregulation in disease therapy, and, finally, strategies that target TAM receptors.

## 2. Structure of TAM Receptors and Their Ligands

All RTKs, including the TAM receptors, have an extracellular domain, a transmembrane domain, and a conserved intracellular kinase domain. The unique conserved sequence KW(I/L)A(I/L)ES in the kinase domain and the unique configuration of two immunoglobulin-like and two fibronectin type III domains within the extracellular domain distinguish TAM receptors from other RTKs [10,11,12].

The two best characterized TAM ligands are the vitamin K-dependent proteins growth arrest-specific 6 (Gas6) and protein S (ProS). The two ligands have a similar domain structure, with an N-terminal γ-carboxyglutamic acid domain that mediates Ca^2+^-dependent binding to a negative charge phosphatidylserine (PtdSer)-presenting membrane, a loop-region, four epidermal growth factor (EGF)-like repeats, and a C-terminal sex hormone-binding globulin (SHBG) domain [13]. The SHBG domain has two globular laminin G-like domains and mediates ligand-receptor binding. Gas6 can bind to all three of the TAM receptors, while ProS only binds to Tyro3 and Mer [14,15]. A recent study, however, showed that ProS can physically bind and activate Axl in glioma sphere cultures [16]. Abboud-Jarrous et al. [17] found that ProS can modulate *Axl* transcription and expression in oral squamous cell carcinoma (OSCC) cell lines. ProS has a thrombin cleavage site and is a plasma glycoprotein that plays a critical negative regulatory role in blood coagulation [18,19,20].

Other TAM ligands are galectin-3 and tubby, which bind to Mer, and tubby-like protein 1, which can bind to all three receptors [21,22]. These ligands were identified to facilitate phagocytosis in retinal pigment epithelium and macrophages. Double knockout of ProS and Gas6 ligands leads to loss of Mer-dependent retinal pigment epithelium phagocytosis in mice [23]. The presence of these ligands (galectin-3, tubby, and tubby-like protein 1) therefore do not restore normal function in retinal pigment epithelium.

The binding of ligand to receptor leads to the formation of a tetrameric complex with a 2:2 stoichiometry [24]. Maximum activation of the receptor requires both the binding by the ligand and the presence of PtdSer-presenting membrane (such as apoptotic cells, enveloped virus, or PtdSer liposomes) [15]. Activation leads to autophosphorylation of tyrosine residues adjacent to conserved sequence in the cytosolic kinase domain. This in turn increases the catalytic efficiency, leading to recruitment and phosphorylation of several signaling molecules with Src homology-2 (SH2), protein tyrosine binding (PTB), and other phosphotyrosine-binding domains [1].

Activation of TAM receptors is linked to several signal transduction pathways such as phosphoinositide 3 kinase (PI3K)/Akt, mitogen-activated protein kinase (MAP kinase), nuclear factor κ-light-chain-enhancer of activated B cells (NF-κB), signal transducer and activator of transcription protein (STAT), phospholipase c-γ (PLC-γ), growth factor receptor-bound protein 2 (Grb2), Raf-1, extracellular-signal-regulated kinase (ERK) and others [25,26,27,28,29,30,31,32,33,34].

## 3. Biological Functions

The three TAM receptors are differentially expressed in different tissue types. Tyro3 is expressed in breast, kidney, lung, testis, osteoclasts, ovary, retina, monocytes, macrophages, platelets [2,35,36,37,38,39,40] and adult central nervous system (CNS) tissue, in particular the cerebral cortex, hippocampal neurons, amygdala, cerebellum, and olfactory bulbs [37,41,42,43]. Axl is expressed near-ubiquitously [10] in various organs including the adult brain (hippocampus and cerebellum), testis, breast, bone marrow stromal cells, platelets, peripheral monocytes, and macrophages [2,35,38,39,44,45]. Mer expression has been reported in the brain, heart, kidney, lung, ovary, prostate, retina, skeletal muscle, testis, and hematopoietic lineages (peripheral blood and bone marrow mononuclear cells, platelets, monocytes, macrophages, dendritic cells, natural killer (NK) cells, and megakaryocytes) [2,11,35,38,39,40]. Even though some cell types such as spermatids, spermatocytes, B cells and T cells are severely affected by the loss of TAM receptors, they do not express these receptors.

TAM signaling pathways play an essential role in hemostasis by stabilizing platelets, regulating inflammation, and promoting phagocytosis of apoptotic cells and cellular debris, as well as maintaining vascular smooth-muscle homeostasis [28,46,47,48,49,50,51].

**Figure 1:**
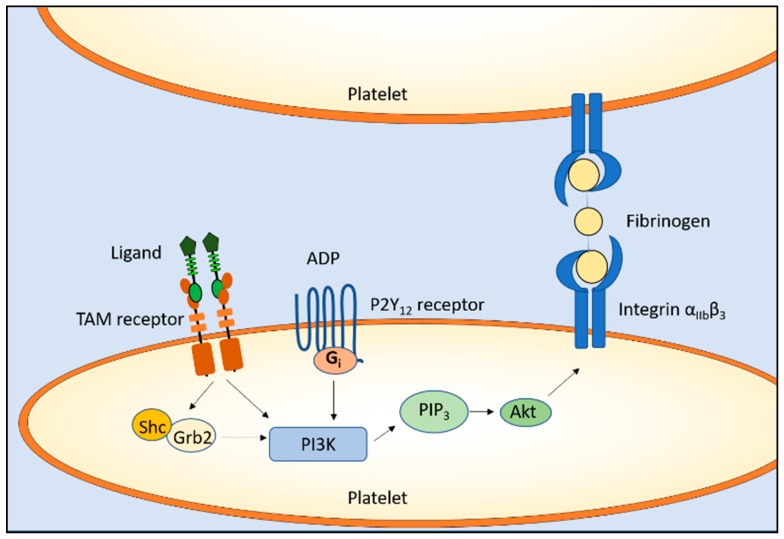
Function of Tyro3, Axl, and Mer (TAM) receptors in hemostasis. In platelets, the activation of TAM receptors and the adenosine diphosphate (ADP) receptor (P2Y_12_) leads to the phosphorylation of phosphoinositide 3 kinase (PI3K) and Akt, resulting in persistent activation of the fibrinogen receptor integrin α_IIb_β_3_ and leading to thrombogenesis and platelet stabilization.

The TAM receptor/ligand complex, together with adenosine diphosphate (ADP) receptor P2Y_12_, leads to PI3K and Akt phosphorylation. This results in persistent activation of the fibrinogen receptor integrin α_IIb_β_3_, leading to thrombogenesis and platelet stabilization [52,53,54] (Figure 1).

Cell death by apoptosis is necessary in many biological processes such as tissue development, homeostasis, lymphocyte maturation, and pathological responses to inflammation. Phagocytosis to clear the apoptotic cells and cellular debris is critical in avoiding tissue necrosis and the release of intracellular content that leads to inflammation and autoantibody production. Loss of TAM receptor function results in a multitude of autoimmune diseases including rheumatoid arthritis and lupus, that are the result of failure to clear apoptotic cells [2,3,5,28]. Macrophage and dendritic cells are responsible for TAM receptor-mediated phagocytosis of apoptotic cells [50]. TAM receptors function as bridges between phagocytes and apoptotic cells that they engulf, with the receptor located on the phagocyte and the TAM ligand bound to the PtdSer-presenting membrane of the apoptotic cell [49].

Besides the role of phagocytosis in immune response, TAM-dependent phagocytosis is also crucial for mediating clearance of apoptotic cells and cell fragments in a variety of other tissues. In the testes, TAM-dependent phagocytosis by Sertoli cells is essential for clearing apoptotic germ line cells and residual bodies of sperm during spermatogenesis [55]. In the retina, retinal pigment epithelial cells are specialized phagocytes that remove used photoreceptor outer segments, preventing accumulation of phototransduction waste [23,56,57,58,59]. A total of 79 genetic variations in the *Mer* gene has been linked to inherited retinal degenerative diseases that result from the failure to remove the photoreceptor outer segment [60]. In the CNS, astrocytes express Mer, which plays a role in clearing debris and remodeling of synapses [61]. After nerve injury, Schwann cells assist in the clearing of myelin debris in an Axl/Mer-dependent manner [62]. Recent studies have reported that Axl and Mer mediate the removal of apoptotic eosinophils to clear allergic airway inflammation and may play a role in asthma [63,64].

**Figure 2:**
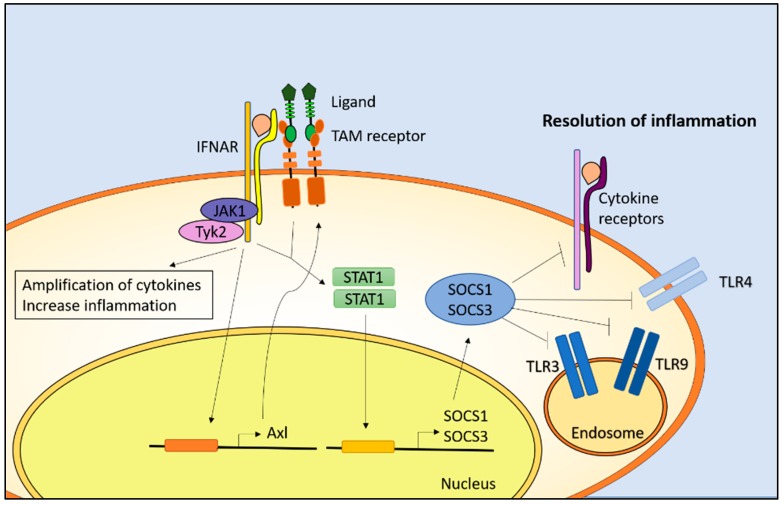
Activation of TAM receptors in dendritic cells down regulate inflammation. Activation of the type I interferon receptor (IFNAR) leads to cytokine amplification, a subsequent increase in inflammation, and the upregulation of *Axl* transcription. The association of the TAM/ligand complex with IFNAR leads to the transcription of SOCS1 and SOCS3. SOCS1 and SOCS3 inhibit the TLR3, TLR4, and TLR9 pathways as well as cytokine receptors to resolve inflammation. TLR: toll-like receptor; JAK: Janus kinases; STAT: signal transducer and activator of transcription protein; SOCS: suppressor of cytokine signaling.

TAM receptors and their ligands function at the interface of the adaptive and innate immune response [65]. The innate immune response is the first-line response against pathogens and leads to the activation of the adaptive immune response. It is crucial that the magnitude and length of the innate immune response is tightly controlled since an overactive immune response can lead to chronic inflammation and autoimmune diseases [66]. After the activation of the innate immune response by pathogens, type I interferon is upregulated. This activates the type I interferon receptor (IFNAR)/Janus kinases (JAK)/STAT pathway that not only increases cytokine production, but also *Axl* transcription [67]. Dendritic cells play an essential role in presenting antigens on their surface, leading to the activation of T helper cells and the engagement of the adaptive immune response. Upon activation, T cells express the TAM ligand ProS [65]. Additionally, ProS is upregulated in macrophages [68]. The TAM receptor/ligand complex associates with activated IFNARs, leading to the transcription of suppressor of cytokine signaling 1 (SOCS1) and SOCS3 [28]. SOCS1 and SOCS3 inhibit toll-like receptors (TLRs) and type I IFNAR/JAK/STAT-dependent upregulation of proinflammatory molecules [69] (Figure 2). The interaction between activated TAM receptors and IFNAR leads to the inhibition of multiple points in the TLR signaling cascade. This includes the down regulation of TLR3-, TLR4-, and TLR9-induced activation of MAP kinase and NF-κB, as well as the inhibition of the TLR-induced ubiquitylation and activation of TNF receptor-associated factors (TRAF6 and TRAF3) [28]. TAM receptors and their ligands protect against autoimmune diseases by down regulating the innate immune response and inflammation.

**Figure 3:**
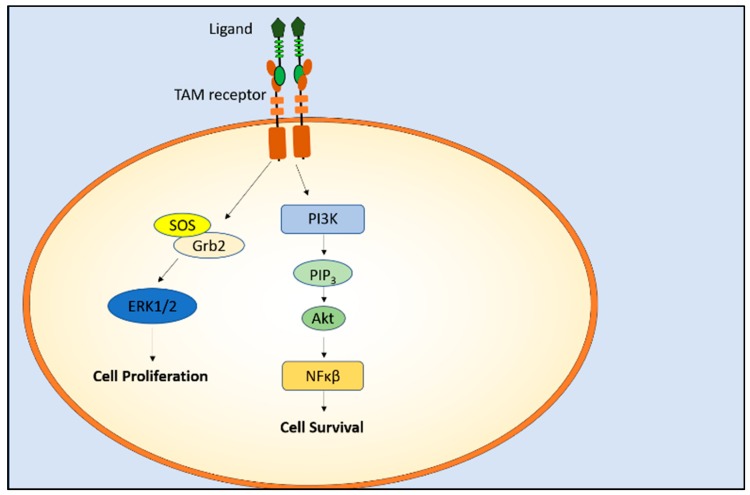
TAM receptor activation results in cell proliferation and survival that are essential for normal neural development. Activation of the PI3K/Akt/NF-κB pathway leads to cell survival, while the activation of SOS/ growth factor receptor-bound protein (Grb2) / extracellular-signal-regulated kinase (ERK) pathway leads to cell proliferation. NF-κB: nuclear factor κ-light-chain-enhancer of activated B cells.

The role of TAM receptors and their ligands in cell proliferation, survival, and migration is vital in neural development and myelination (reviewed in [70]). Upon activation of Axl, Grb2 and SOS are recruited, which leads to the activation of ERK, cell migration and proliferation [71]. Additionally, activated Axl and Mer lead to the phosphorylation of PI3K and Akt that activate the NF-κB signaling pathway leading to cell survival [29,72] (Figure 3).

Alternative splicing [10,73,74,75] or proteolytic cleavage by metalloproteinase [76,77] can result in soluble circulating TAM receptors (sTyro3, sAxl, and sMer). Alternative splicing influences the location and function of receptors, while proteolytic cleavage inactivates receptors. Circulating receptors may also bind ligands to act as a decoy [78]. Circulating receptors can influence signaling pathways and impair phagocytosis of monocytes/macrophage [50].

Abnormal levels of TAM receptors are linked to several disease states. The loss of TAM receptor function results in autoimmune diseases, while increased TAM expression leads to chemo-resistance, metastasis, and high mortality in cancer patients. In this review we discuss both theses aspects, as they are two sides of the same coin.

## 4. TAM Receptors and Autoimmune Diseases

Autoimmunity is the result of the organism’s failure to recognize its own constituent parts as belonging to itself, leading to immune response against its own cells and tissues. As previously described, phagocytes must clear apoptotic cells. Failure to do this exposes the immune system to the accumulated intracellular components, including nuclear antigens. This can lead to the formation of immune complexes and autoreactive B cells that produce autoantibodies [79,80]. The aforementioned process, together with enhanced activation of dendritic cells and the type I interferon (IFN) response, contribute to autoimmunity [81,82,83]. The loss of TAM signaling prevents optimal phagocytosis of apoptotic cells, thus leading to an overactive inflammatory response that can cause autoimmunity [50,84]. Mice lacking TAM receptors display traits of systemic autoimmunity, including high titers of circulating anti-chromatin, anti-double stranded DNA, anti-single stranded DNA, and anti-phospholipid antibodies [39,85].

The role of TAM receptors in autoimmune disease has been studied in diseases such as systemic lupus erythematosus (SLE) and multiple sclerosis (MS). SLE is a chronic systemic autoimmune disease that affects multiple organs including the heart, joints, skin, lungs, blood vessels, liver, kidneys, and nervous system [80,86]. The course of the disease is unpredictable, with periods of illness alternating with remissions [87].

Reduced TAM signaling has been described as contributing to the development of SLE (reviewed in [88]). The accumulation of apoptotic cells in lymph nodes of SLE patients is associated with ProS levels in circulation [81,89,90]. The role of TAM receptors in scavenging apoptotic cells is consistent with the correlation between the decreased ProS levels and lupus disease activity [90]. ProS has a more important function in SLE patients than Gas6, since Gas6 forms a stable complex with sAxl in human serum. In serum, the molar concentration of sAxl is higher than that of Gas6, blocking the activity of the ligand [91]. Gas6 activates the recycling of Axl in the cell membrane [92]. Gas6 activation of Axl and Mer has been shown to downregulate the inflammatory innate response for several of its cellular components, including dendritic cells, NK cells and macrophages [93].

Ballantine et al. [94] investigated the role of Mer and the three soluble TAM receptors in the phagocytosis potential of monocytes and macrophages in juvenile SLE (JSLE). They observed significantly increased levels of sMer, sAxl, and sTyro3 in JSLE plasma, with reduced levels of Mer receptors on JSLE monocytes. Mer is also the main receptor used by mouse macrophages to phagocytose apoptotic cells [50]. Knockout of Mer abolished phagocytosis of apoptotic cells, whereas Tyro3 or Axl knockout reduced macrophage phagocytosis by about 50% in mice [50]. The increased cleavage of the Mer, and consequently increased sMer, has a major impact on the phagocytosis rate within JSLE [94]. sMer inhibits phagocytosis in vitro [78] and correlates with disease activity in adult SLE [95]. This may be the cause of significant deficit in apoptotic cell clearance found in adult SLE.

MS is an autoimmune inflammatory disease resulting from an autoimmune attack against CNS myelin antigens. This causes damage to myelin and leads to neurological dysfunction. The disease mainly affects young adult women between the ages 20 and 40 years [96,97,98]. In the CNS, all three TAM receptors are expressed [99].

The Gas6/TAM complex is important to brain development during embryogenesis. It also plays a role in clearing cellular and myelin debris from inflammatory demyelination. This is an important initial step to recover myelin fibers [100]. Additionally, the Gas6/TAM complex promotes survival of neuron and glial cell in the CNS, contributing to the process of myelination [100].

Cuprizone (bis-cyclohexanone-oxaldihydrazone)-treated mice are used as a model to study MS. Cuprizone causes toxic demyelination without altering the blood/brain barrier. In cuprizone damaged tissue, oligodendrocytes and microglial/macrophages fail to accumulate [100,101,102]. In mice, cuprizone treatment results in decreased Tyro3 and increased *Gas6*, *Axl*, and *Mer* transcription [100,103]. More severe demyelination, a greater reduction in oligodendrocyte number, and an overactivation of microglia was found in mice lacking Gas6 [103]. Gas6 was demonstrated to promote the generation of oligodendrocytes and myelin in the white matter of the adult mouse optic nerve, indicating an important role for Gas6 in promoting CNS repair after demyelination and is a potential target for therapeutic approaches for MS [104].

## 5. TAM Receptors and Cancer

In the last decade, the number of reports describing the role of TAM receptors in cancer progression and development, as well as their role in therapeutic strategies has increased considerably. Since their initial description, TAM receptors have been linked to several types of cancer. As with Mer and Axl, information regarding Tyro3 and its role in human cancer is scarce. It is upregulated in leukemia [105], thyroid cancer [106], metastatic colorectal tumors [107] and melanoma [108]. On the other hand, Mer has been described as being upregulated in several types of cancer, such as non-small cell lung cancer (NSCLC) [109], melanoma [109,110], acute myeloid leukemia (AML) [111], and schwannoma [112]. Among members of TAM receptors, Axl has been extensively studied in several types of cancers [113]. Reports have demonstrated Axl upregulation in tumors such as prostate [114,115,116,117], ovarian [118,119,120,121,122,123], NSCLC [124,125], OSCC [126,127,128], osteosarcoma [129,130,131], AML [132], schwannoma [112], glioma [16,133,134,135], and thyroid cancer cell lines [106,136].

It is important to note that overexpression of TAMs can drive conventional oncogenic signaling and survival pathways in cancers [105], while also playing an important role in epithelial to mesenchymal transition (EMT) and metastasis [137,138]. Axl activation has been linked to signaling cascades closely related to progression and development of tumors. Furthermore, Axl protects cells from apoptosis [139], a characteristic that has been described in different types of cancers. A strong positive correlation between phosphorylated Axl and matrix metalloproteinase (MMP)-9 expression has been described in osteosarcoma patients and in breast cancer models [140,141]. It is important to highlight that proteins of the matrix metalloproteinase (MMP) family are involved in the breakdown of the extracellular matrix which is closely associated with metastatic potential.

The role of TAM receptor Axl has been described in both prostate cancer and OSCC [116,126]. In prostate cancer, Paccez et al. [116] demonstrated that Axl levels are expressively higher in both androgen-insensitive and androgen-sensitive prostate carcinoma cell lines. Axl was found to be upregulated in about half of human prostate tumors. Its blockage inhibited proliferation, invasion and migration of prostate cancer cell lines, as well as reduced tumor formation in a xenograft mice model. Axl inhibition leads to inactivation of the NF-κB pathway through inhibition of Akt and inhibitor of NF-κB kinase subunit alpha (IKKα) activity, which, in turn, blocks the interleukin 6 (IL-6)/STAT-3 signaling pathway [116].

In addition, Paccez et al. [126] demonstrated that Axl is overexpressed in OSCC cell lines and human tumor samples in the South African population. Repression of Axl expression leads to Akt/NF-κB inhibition and the induction of glycogen synthase kinase 3β (GSK3β) activity, resulting in the loss of mesenchymal markers and the induction of epithelial markers [126]. These findings have an impact on the biology of both prostate and esophageal cancers, supporting the notion that Axl may be employed as a therapeutic target for treatment of these diseases.

Recently, another pathway related to tumor progression has been described. TAM receptors are expressed on infiltrating myeloid-suppressor cells, macrophages and NK cells that contribute to tumor progression since these cells may affect immune escape [8,142,143]. Infiltration of Mer-expressing NK and M2 macrophages is associated with suppression of anti-tumor immune responses, demonstrating that TAM receptors are not only involved with the deregulating pathway through conventional tumorigenesis, but may also play a role in inhibiting signals, promoting tolerance and immune suppression in the tumor microenvironment [144]. The immune checkpoint molecule, programmed cell death ligand 1 (PD-L1), is upregulated in tumor cells by TAM receptors that enable immune escape [145]. Some tumors secrete ProS, that can bind to Mer and Tyro3 on resident macrophages, inhibiting their capacity to assume an antitumor phenotype [146].

Tumor heterogeneity states the presence of different cell populations (i.e., cells with distinct genotypes and phenotypes). These subpopulations of cells may display different biological behaviors within a primary tumor and its metastases, or between tumors of the same histopathological subtype (intra- and inter-tumor, respectively). In fact, heterogeneous tumors show partial therapy responses which may allow the emergence of drug-resistant clones. In the last decade, a common feature of drug-resistant cancer is the expression of high levels of Axl [109,147,148,149,150]. Activation of tyrosine kinases such as Axl, MET, and epidermal growth factor receptor (EGFR), lead to EMT, which is linked to the development of drug resistance [151,152,153,154,155,156]. In this context, Axl expression enables resistance to target agents; specifically, inhibitors of other RTKs. Schoumacher and Burbridge [157] describe two interesting mechanisms by which TAM receptors may contribute to drug resistance: (1) maintenance of the same pathway activity via alternative effectors, and (2) activation of distinct signaling networks [157].

Muller et al. [158] and Konieczkowski et al. [159] found that melanoma cells expressing high levels of Axl are resistant to MAP kinase pathway inhibitors [158,159]. Zhang et al. [148] describe Axl upregulation in EGFR inhibitor-refractory lung cancer. There are also reports of upregulation of Axl in anti-HER2-resistant breast cancer, unitinib-resistant renal cell cancer [160], and anaplastic lymphoma kinase (ALK) inhibitor-resistant neuroblastoma [138,161], as well as in head and neck cancer resistant to PI3K inhibitors [149]. In a recent report, Lin et al. [162] demonstrated that Axl mediates docetaxel resistance in prostate cancer. The authors found that overexpression of Axl can induce resistance to docetaxel and that the activation of Axl is independent of Gas6 in docetaxel-resistant prostate cancer cells [162]. Activation of Axl in RTK-resistant cancer such as EGFR may occur due to bypassing of the RTK inhibitor effect. In drug-resistant cancer, a bypass (activation of a secondary RTK) reactivates downstream pathways while the primary oncogene is still inhibited, leading to continued proliferation/survival in the presence of the inhibitor.

Other than simply correlating Axl expression to induction of resistance in cancer, several reports described the ability of Axl to dimerize with other receptors, such as EGFR, MET, and platelet-derived growth factor (PDGFR) [147,163,164]. This heterodimerization allows the triggering of signals that increase proliferation, migration and invasion of cancer cells. Vouri et al. [156] described the interaction between EGFR and Axl. They demonstrated different sets of gene expression patterns regulated by EGFR–EGFR, Axl–Axl, and EGFR–Axl dimerization. For instance, genes promoting invasion were regulated by the EGFR–Axl axis, while the EGFR–EGFR axis regulated the cell cycle and Axl–Axl regulated invasion [156]. In this context, the development of resistance to EGFR-inhibitors may be induced by EGFR–Axl transactivation in order to amplify signals important in inducing cell invasion, but that are not activated by EGFR–EGFR activation.

### Targeting TAM Receptors

As previously discussed, increased activity of TAM receptors, other than activating oncogenic signals, may lead to drug resistance, increase tumor progression and aggressiveness. In this regard, the development of therapy targeting this class of proteins may represent improvement in the treatment outcome of several types of cancer. The development of TAM inhibitors includes: (1) small molecule tyrosine kinase inhibitors, (2) antagonistic monoclonal antibodies, (3) TAM-fragment crystallizable region of antibody (Fc) soluble decoy receptors, and (4) aptamers. Although promising, each of the approaches has positive and negative points that must be considered.

The development of small molecules demonstrated strong inhibitory activity, however, most are not specific to the TAM receptors [165]. The Axl inhibitor BGB324 has been shown to inhibit both Mer and Tyro3 at high concentrations [166], while the Mer inhibitors UNC569 and UNC1666 also have inhibitory effects on Axl and Tyro3 [167,168]. These inhibitors have also been described as possessing off-target effects in other proteins such as vascular endothelial growth factor receptor (VEGFR), Abelson tyrosine-protein kinase (Abl), tunica internal endothelial cell kinase 2 (Tie-2), MET, Fms-like tyrosine kinase (Flt3), and rearranged during transfection (RET) protein [165,167,168].

The development of strategies to inhibit TAM receptors are more prominent using Axl as a model. Some clinical trials involving BGB324 have been employed in NSCLC (NCT02424617 and NCT02922777), AML (NCT02488408), and triple-negative breast cancer (NCT03184558). BGB324, a small molecule inhibitor of Axl developed by Holland et al. [169], blocks Axl-dependent signaling events, reduces the EMT transcriptional regulator Snail, and inhibits angiogenesis and tumor formation [169]. BGB324 can also inhibit survival and proliferation of glioblastoma cell lines in a concentration-dependent manner [170] and increase survival of immunocompromised mice bearing glioma sphere culture-derived mesenchymal glioblastoma-like tumors [16]. BMS-777607, a selective and potent small-molecule MET inhibitor, has similar effects on these cell lines and mice models, as an Axl-inhibitor [171]. Another small molecule named TP-0903 induces apoptosis in primary chronic lymphocytic leukemia (CLL) B-cells by targeting Axl without inhibiting phosphorylated Tyro3, overcoming bone marrow stromal cell protection of the leukemic B-cells [172]. In spite of targeted small molecular inhibitors having good toxicity profiles, patients who enter treatment may develop severe toxicities [173].

The pipeline for the development of specific target inhibitors is costly. Alternatively, natural compounds have been identified as specific TAM inhibitors. Han et al. [174] demonstrated that MTE, water extract of *Marsdenia tenacissima*, is able to restore erlotinib/gefitinib sensitivity in tyrosine kinase inhibitor (TKI)-resistant non-small cell lung cancer cells driven by Axl and MET [174].

Ye et al. [175] developed a monoclonal antibody (YW327.6S2) against Axl, which recognizes the receptor with high affinity, diminishing xenograft tumor growth and potentiating the effect of anti-VEGF treatment [175]. Recently, an antibody–drug conjugate, AXL-107-MMAE, was developed that linked a human Axl antibody with the microtubule-disrupting agent monomethyl auristatin E. Treatment with AXL-107-MMAE has in vivo anti-tumor activity in melanoma, lung, pancreas and cervical cancer xenograft models [176].

Furthermore, Liu et al. [177] described the use of a monoclonal antibody (MAb173), that not only induces Axl degradation in vitro and leads to inhibition of Kaposi sarcoma cell invasion, but also reduces tumor growth, increases tumor cell apoptosis, decreasing the Axl level in in vivo xenograft studies [177]. The same group developed a humanized version of this monoclonal antibody (hMAb173) that can induce renal cell carcinoma apoptosis ex vivo and in vivo [178].

Soluble TAM receptors generated from proteolytic cleavage or alternative splicing can quench available ligands, limiting TAM signaling [179]. Kariolis et al. [180] engineered an Axl decoy receptor that binds Gas6 with high affinity. By neutralizing ligands this decoy could inhibit Gas6/Axl signaling, metastasis, and disease progression in vivo [180].

Aptamers are molecules (stable nucleic acid) that bind targets with high affinity and specificity. In this sense, aptamers may represent alternatives with potentially fewer toxic effects than current standard therapies [181]. The advantages of aptamers are high affinity for its target [182,183] and low sensitivity to endo-nucleases when engineered to include chemical modifications, such as fluoro and phosphorothioate modifications [184].

Cerchia et al. [185] developed an RNA aptamer, GL21.T, specific to inhibiting Axl that also binds to the Tyro3 ectodomain [185]. Kanlikilicer et al. [181] demonstrated that a chemically modified DNA aptamer inhibited Axl in vivo. They also demonstrated reduction in antitumor/metastasis and enhanced the efficacy of chemotherapy (paclitaxel) in ovarian cancer tumor models [181].

## 6. Conclusions

TAM receptors and their ligands play an intricate role in tissue maintenance and development. TAM signaling is highly regulated. Loss of function is associated with disarray in homeostasis that leads to autoimmune diseases, while increased activity is associated with poor prognosis, resistance and metastasis in cancer patients. Inhibition of TAM signaling has shown promise in cancer treatment. TAM inhibitors can restore drug sensitivity, inhibit angiogenesis, reduce tumor growth, and inhibit tumor formation. The consequences of long-term inhibition of TAM signaling are unexplored in the light of disease development due to loss of function. Understanding the role of these receptors in diseases can aid in the development of more rational, therapeutic modalities. The development of targeted TAM therapies for the treatment of cancer without affecting other cells and tissue in which their role is crucial remains paramount.
